# The role and importance of SBRT in prostate cancer

**DOI:** 10.1590/S1677-5538.IBJU.2018.0484

**Published:** 2018

**Authors:** Yasemin Cihan

**Affiliations:** 1 Department of Radiation Oncology, Kayseri Egitim ve Arastirma Hastanesi, Hastane cad Kayseri, Kayseri, Turkey

*To the editor,*

Practical applications in radiotherapy have developed considerably over the past 60 years. Medical imaging, immobilization techniques and advances in computer soft ware programs enable the use of broad-based stereotactic body radiotherapy (SBRT). SBRT is a special type of external radiation therapy that is irradiated with a high radiation dose of 1 to 5 fractions with smaller safety margins than the target conventional irradiation. Although different from traditional radiobiological concepts, SBRT is a promising treatment model with a high local control rate, low normal tissue toxicity, and short treatment duration compared with conventional radiotherapy. Stereotactic radiotherapy can be used in various parts of the body due to noninvasive stabilization methods and the availability of taking target images during treatment. It has been started to be used in primer lung cancers and lung metastases, primary liver cancers and liver metastases, pancreatic cancers, prostate cancer, recurrent head and neck cancer, recurrent gynecologic cancers and many other types of cancer and in different regions (1-5).

Hypofractional radiotherapy to the prostate is based on modern radiobiology knowledge and advances in SBRT. Many trials of hypofractional administration in prostate cancer have demonstrated that hypofractionation treatment provides the same or better tumor control than normal fraction therapy, while late and early toxicity remains unchanged in normal healthy tissue. According to clinical data, large fraction doses are biologically superior in prostate cancer to small fraction doses. Due to the low α/β ratio (1.4-3 Gy) of tumor cells in prostate cancer, high doses can be achieved. Therapeutic rate is also increased with hypofractionational application (2, 3, 5-6). Recently, SBRT has been started to be used in the treatment of prostate cancer in curative, salvage and boost treatments in the literature. Studies presenting the specific complications and success rates of all these applications have begun to be published. According to the results of the studies on the efficacy of SBRT treatment for prostate cancer treatment, biochemical relapse free survival of 90-100% with a median follow up of 5 years or more was reported (1-4). In a study conducted by Katz, in low-risk prostate cancer SBRT was 35 Gy-36.25 (equivalent dose of 90-95 Gy at 1.8 Gy per fraction, or 200-212 Gy BED). It has been shown that Gy dose is an effective and low toxic treatment in the early period. Again, in this study it was stated that dosing above 35 Gy resulted in more toxicity than clinical benefit (6). Koskela et al. investigated the efficacy of SBRT in high-risk group. They emphasized that genitourinary or rectal toxicity wasn't found at acute grade 3 and above. The rates of intermediate-term grade 3 genitourinary, rectal and infectious toxicity were low. They stated that PSA control was better in the low- and moderate-risk group (7). Janowski et al reviewed the efficacy of SBRT in patients with high prostate volume. This study investigated the efficacy and toxicity profile of SBRT in 57 prostate cancer patients with low, moderate, and high risk groups and prostate volume ≥50 cm^3^. SBRT Cyberknife (Accuray) device was used with doses of 35-36.25 Gy in 5 fractions. The 2-year actuarial incidence rates of genitourinary and gastrointestinal toxicity ≥ grade 2 were 49.1% and 1.8%, respectively. They reported that SBRT is more reliable and effective than brachytherapy and conventional radiotherapy (RT). Another feature of this study is the evaluation of symptom flare of late gastrointestinal toxicity due to SBRT and the improvement with conservative treatment (1). Mbeutcha et al compared high-dose brachytherapy in post radiation salvage therapy in patients with prostate cancer previously treated with RT with SBRT. They emphasized that both treatments were effective in the treatment of salvage (2). Fuller et al followedup recurrent prostate cancer patients treated with SBRT in 34 Gy / 5 fraction for median 24 months. They reported 2-year biochemical-free survival of 82% with only 7% of grade 3-4 urinary toxicity and no severe digestive toxicity (3). Paydar et al investigated whether boost therapy with SBRT (19.5Gy in three fractions) was as effective and reliable as brachytherapy in patients who received intense-modulated radiation therapy (IMRT) (45-50.4 Gy). Inpatients they followed up median 4.2years, cumulative late ≥ grade 2 and ≥ grade 3 genitourinus toxicities were observed respectively in 40 and 6% of the patients. Overall modest rates of gastrointestinal toxicity were with a 12% cumulative incidence of late ≥grade 2GI toxicity, 7% late ≥ grade 2 rectal bleeding, and 1% late grade 3 bleeding. They emphasized that the side effect of SBRT boost treatment after IMRT is less and more reliable (8).

In conclusion, the data that do not have sufficient maturity and follow-up period suggest that today a gold standard for effective treatment of SBRT in prostate cancer treatment is not yet available. However, because of the characteristics of SBRT, it is seen that it can be used as the prostate therapy and as an alternative to effective treatment methods. There is a need for studies to be conducted in this regard.
